# Pictorial essay: B-scan ultrasonography in ocular abnormalities

**DOI:** 10.4103/0971-3026.50827

**Published:** 2009-05

**Authors:** VD Aironi, SG Gandage

**Affiliations:** Department of Radiodiagnosis, Rural Medical College, Loni 413736, Rahata, Ahmednagar, India

**Keywords:** B-scan, ocular pathologies, retinal detachment

## Abstract

B-scan ultrasonography (USG) is a simple, noninvasive tool for diagnosing lesions of the posterior segment of the eyeball. Common conditions such as cataract, vitreous degeneration, retinal detachment, ocular trauma, choroidal melanoma, and retinoblastoma can be accurately evaluated with this modality. B-scan USG is cost-effective, which is an important consideration in the rural setting. In addition, it is noninvasive and easily available and the results are reproducible.

## Introduction

The eyeball's fluid content and its superficial position make it ideally suited for examination with ultrasonography (USG).[1,2] USG is the only practical method for obtaining images of the posterior segment of the eye when the light-conducting media are opaque. It is the most useful investigation prior to vitrectomy.[1] In this pictorial essay, we highlight the various conditions that can be evaluated by B-scan USG.

## Material and Methods

All images presented in this article were obtained using a standard USG machine (Logiq 400 Pro series, Wipro GE, Bangalore, India) equipped with a 7.5–10 MHz real-time high-frequency probe with the contact method. The probe was placed over the closed eyelid after application of coupling gel.

### Normal B-scan and Anatomy [Figures 1, 2]

**Figure 1:**
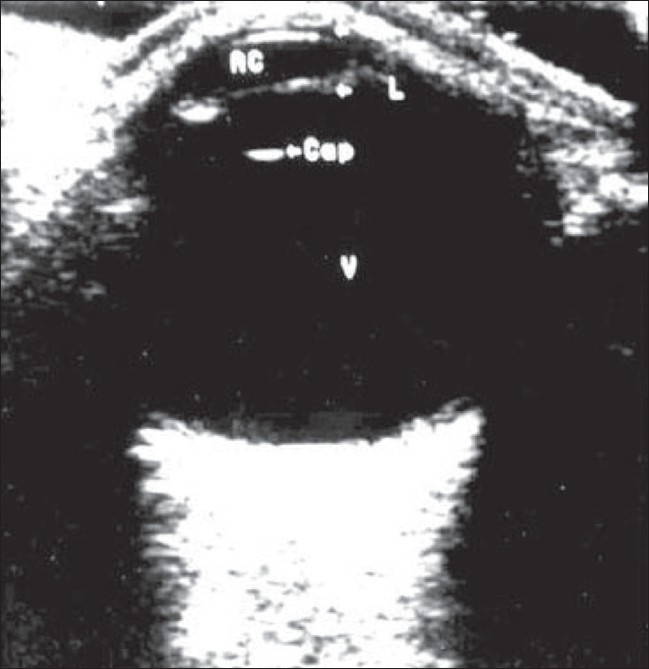
Normal anatomy. B-scan of the normal eyeball shows normal clear vitreous (v) in the posterior segment, with the posterior lens capsular echo (labeled as ‘cap’) anteriorly

**Figure 2:**
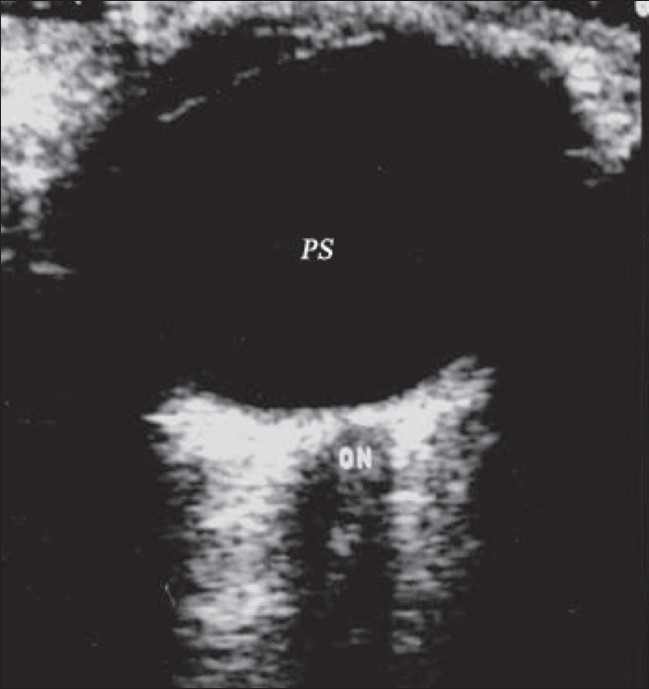
Normal anatomy. B-scan reveals a normal eyeball with the optic nerve (ON) passing through the retrobulbar fat. Normal posterior segment (PS) contains clear anechoic vitreous

The human eye, with its superficial position and its fluid-filled structure, is ideally suited for examination by USG. The eyeball has a transparent anterior segment and an opaque posterior segment containing the echolucent vitreous.[1–5] The iris diaphragm divides the anterior segment, which is filled with aqueous humor, into two chambers. The eyeball has three coats: the sclera, choroid, and retina. The lens is a transparent, biconvex body situated behind the iris. On B-scan, normal clear vitreous is seen in the posterior segment with the echo of the posterior lens capsule seen anteriorly. The axial length of the normal adult eye is 24 mm.[1,2,4]

On B-scan of the normal eyeball, the optic nerve can be seen passing through the retrobulbar fat. The retrobulbar fat is echogenic, and the optic nerve is seen as a hypoechoic tubular structure extending from the posterior pole of the eyeball toward the orbital apex.[1–4] The extraocular muscles can be identified on a B-scan, especially the medial and lateral recti, on a horizontal scan.

### Various ocular conditions detected by B-scan Cataract

**Figure 3:**
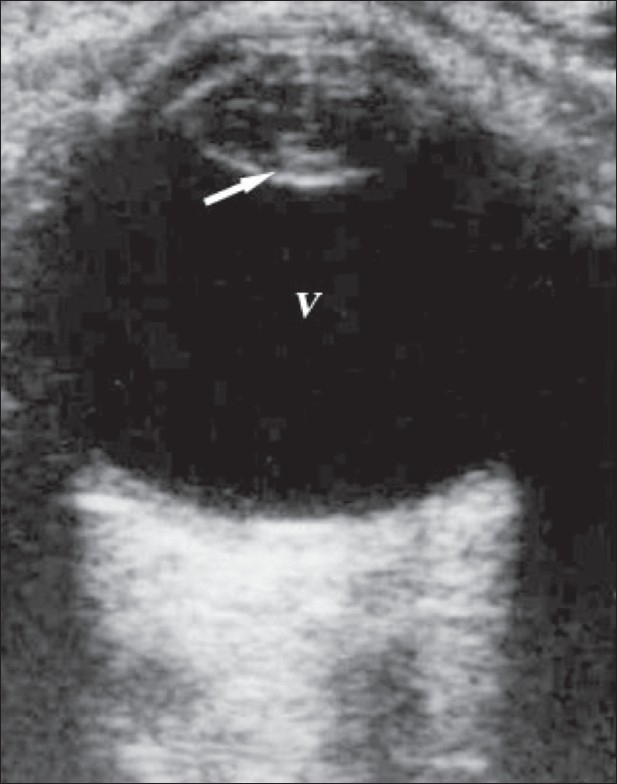
Cataract. This is a 65-year-old man with a cataract. USG reveals reflective material in the lens substance, with a marked posterior capsular echo (arrow). The whole lens is seen with a totally opaque cortex. This highly reflective lens is suggestive of cataract

**Figure 4:**
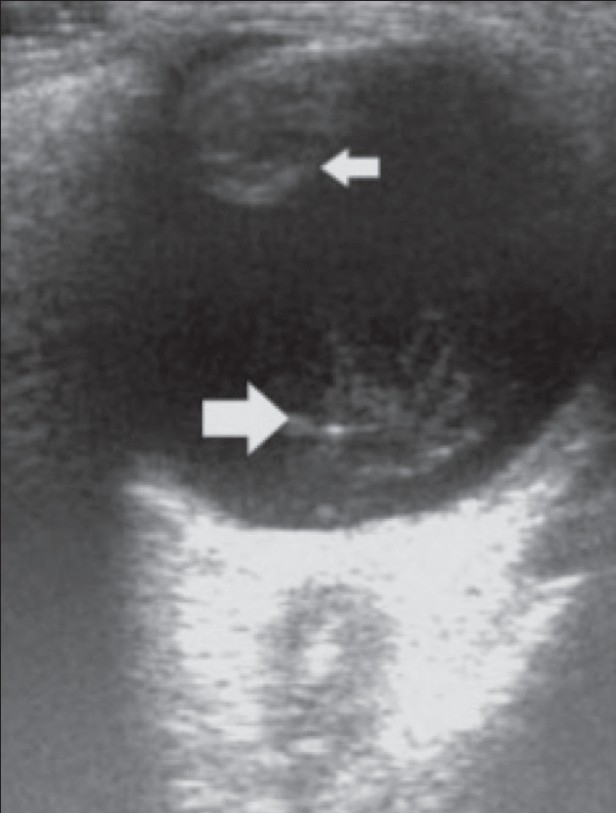
Cataract. B-scan reveals a cataractous lens (small arrow) associated with organized vitreous hemorrhage (big arrow) in an elderly, diabetic patient

Cataract is a degenerative disease of the lens that is usually seen in the older age-groups. It can sometimes be secondary to trauma, when the lens becomes opaque due to deposition of reflective material beneath the lens capsule. On B-scan, an immature cataract shows scattered opacities separated by clear zones. In a mature cataract, the lens has a completely opaque cortex and is seen as a very dense structure[1–4] [Figures 3 and 4].

### Vitreous degeneration

**Figure 5:**
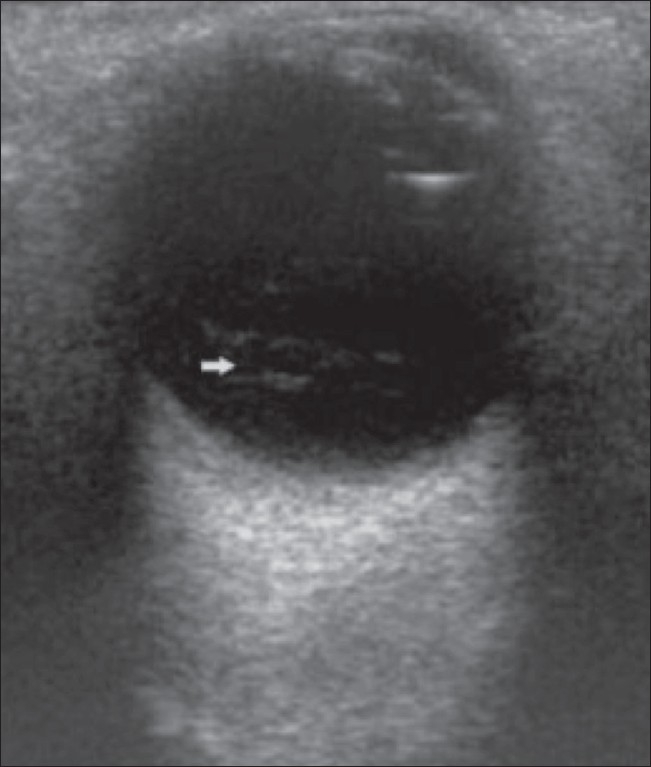
Vitreous degeneration. This 60-year-old woman with diabetes mellitus had vitreous degeneration in the right eye. B-scan reveals multiple hyperreflective, mobile foci (arrow) within the vitreous chamber. After-movements were seen on the dynamic scan

In vitreous degeneration, the liquefied vitreous contains cholesterol crystals that move with eye movements. B-scan reveals multiple hyperreflective mobile foci within the vitreous chamber that show after-movements on a dynamic scan. This is also known as synchysis scintillans. There is no reduction of visual acuity in this condition. It is often bilateral and secondary to longstanding uveitis or may follow vitreous hemorrhage[1–4] [Figure 5].

### Classic retinal detachment

**Figure 6:**
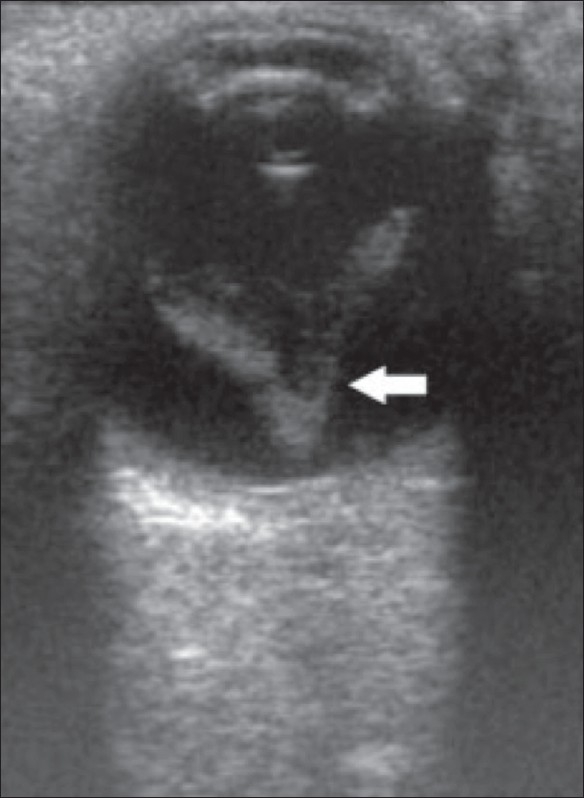
Retinal detachment. B-scan reveals a classic total RD (arrow) in a 60-year-old man who came with loss of vision in his right eye. The retina has a funnel-shaped appearance due to firm attachment at the ora serrata anteriorly and the optic nerve head posteriorly. Dynamic study showed reduced retinal mobility. The retinal leaves are thick

Retinal detachment (RD) is usually due to a break or tear in the retina; it may also be caused by vitreoretinal traction due to contracting membranes or because of subretinal exudates. The detached retina is usually attached to the firm anchoring points of the ora serrata anteriorly and the optic nerve head posteriorly and, consequently, a total RD shows a funnel shape.[1–4] Dynamic scan may reveal an undulating motion of the retinal membrane, particularly in a recent RD. With B-scan, it is possible to diagnose RD early so that reparative surgery can be carried out to seal the retinal tear using laser or cryotherapy[1–4] [Figure 6].

**Figure 7 (A,B):**
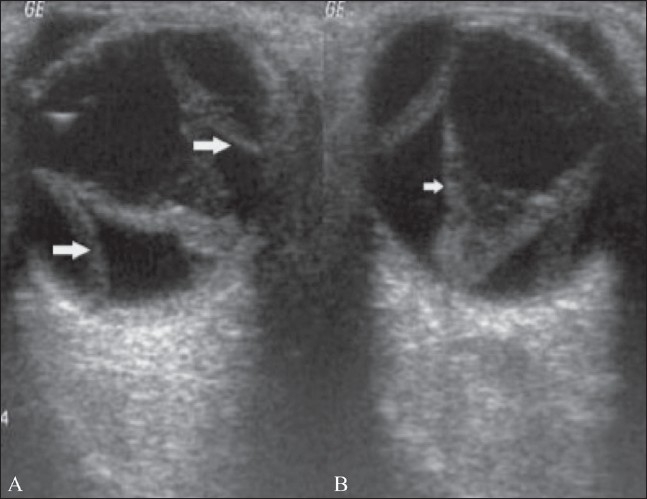
Retinal and choroidal detachment. B-scan reveals an RD (small arrow in B) in association with a choroidal detachment (big arrows in A)

RD is sometimes seen in association with choroidal detachment [Figure 7]. In a choroidal detachment, B-scan shows fluid in the suprachoroidal space; the choroid is attached anteriorly to the ciliary body (scleral spur) and posteriorly at the exit foramina of the vortex veins. It may be secondary to trauma or surgery or may even occur spontaneously.[1,4]

### RD with persistent hyperplastic primary vitreous (PHPV)

**Figure 8:**
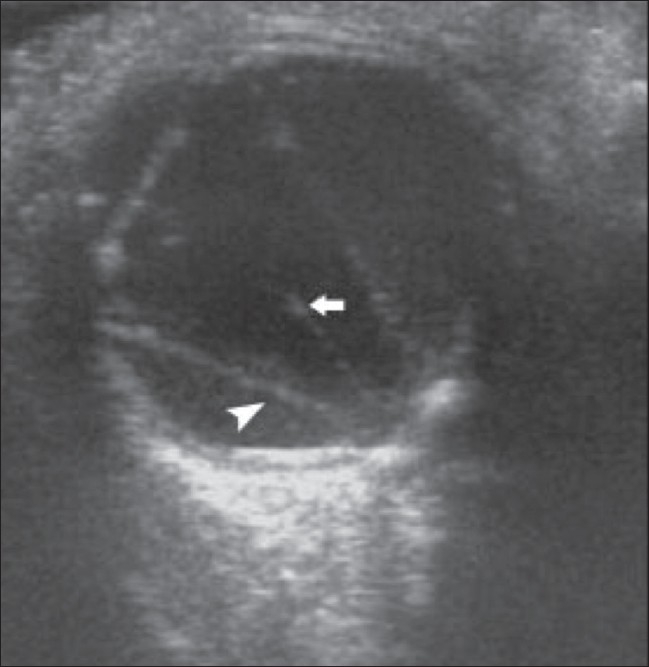
Retinal detachment with PHPV. B-scan reveals a funnelshaped RD (arrowhead) along with a persistent central hyaloid artery (arrow) in a 1-year-old child

In persistent hyperplastic primary vitreous a funnel-shaped RD is seen associated with a persistent central hyaloid artery [Figure 8].

### Persistent primary hyperplastic vitreous

**Figure 9:**
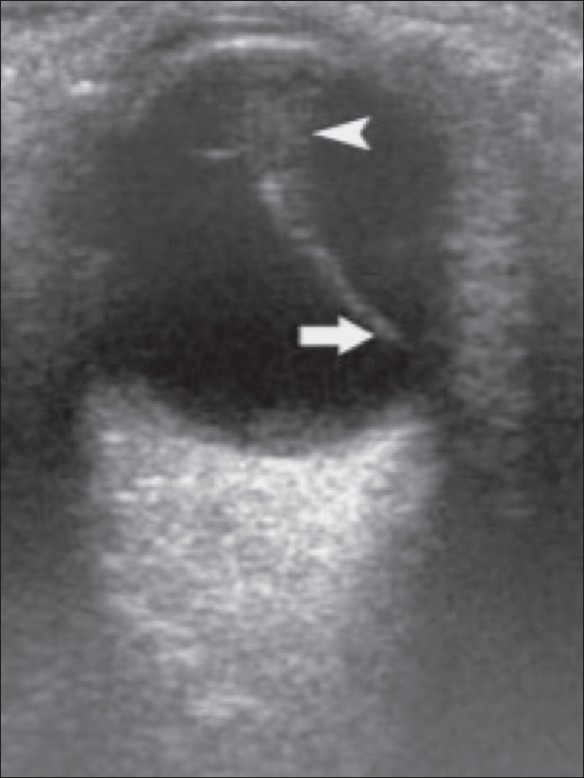
PHPV. B-scan in a 5-month-old infant with leukocoria shows presence of a dense membrane containing the hyaloid artery (arrow) extending from the retrolental region to the optic disc. There is a retrolental mass (arrowhead)

PHPV is a serious unilateral disorder of the vitreous that is seen in childhood. It presents as leukocoria (white pupil). There is failure of regression of the primary vitreous.[1,2,4] The primary vitreous persists in a microphthalmic eye and B-scan shows a retrolental membrane, which may be dense; there is a persistent hyaloid artery extending from the retrolental region to the optic disc[1–4] [Figure 9].

### Retinopathy of prematurity

**Figure 10:**
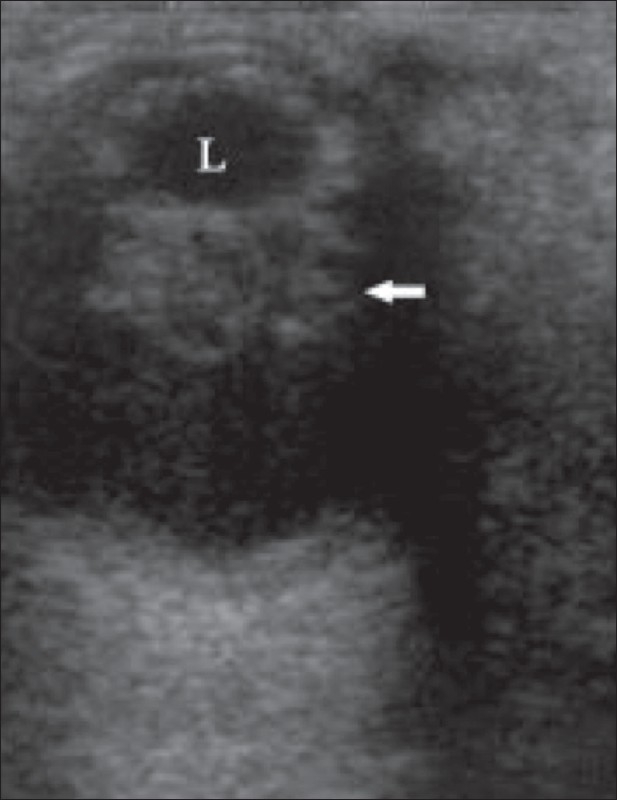
Retrolental fibroplasia. A 6-month-old infant with leukocoria had retinopathy of prematurity (retrolental fibroplasia). A retrolental mass (arrow) is seen posterior to the lens (L) on the B-scan

Retinopathy of prematurity is a bilateral condition that is associated with a history of prematurity and oxygen therapy in the postnatal period. This leads to the occurrence of retrolental fibroplasia, with development of dense retrolental membranes,[1,2,4] a result of neovascularization from the retinal periphery leading to fibrotic changes in the anterior vitreous. The eyeballs are normal in size[1–4] [Figure 10].

### Choroidal melanoma

**Figure 11:**
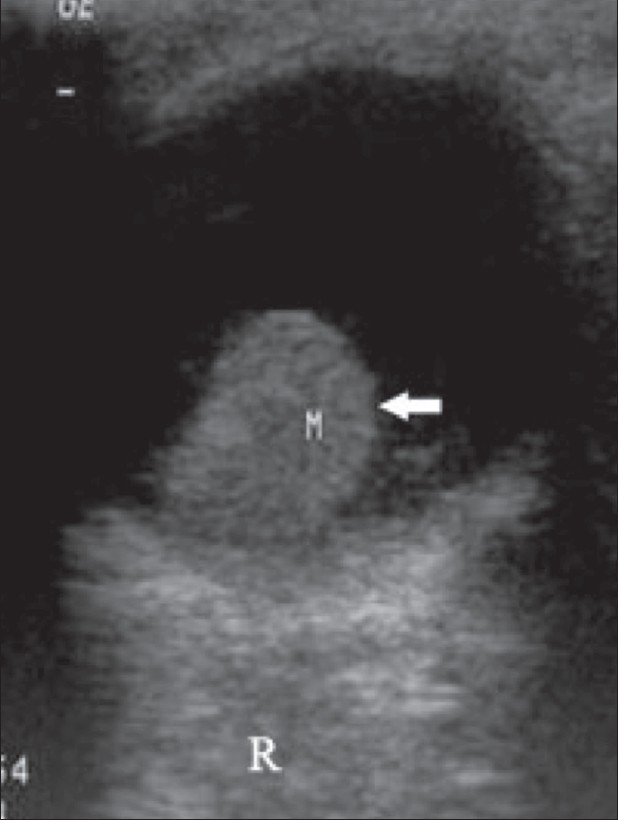
Choroidal melanoma. USG reveals a lenticular-shaped mass (arrow) deeply embedded in, and arising from, the choroid in an adult woman with a history of gradual loss of vision in her right eye (R). The mass is moderately reflective a sign suggestive of choroidal melanoma

**Figure 12:**
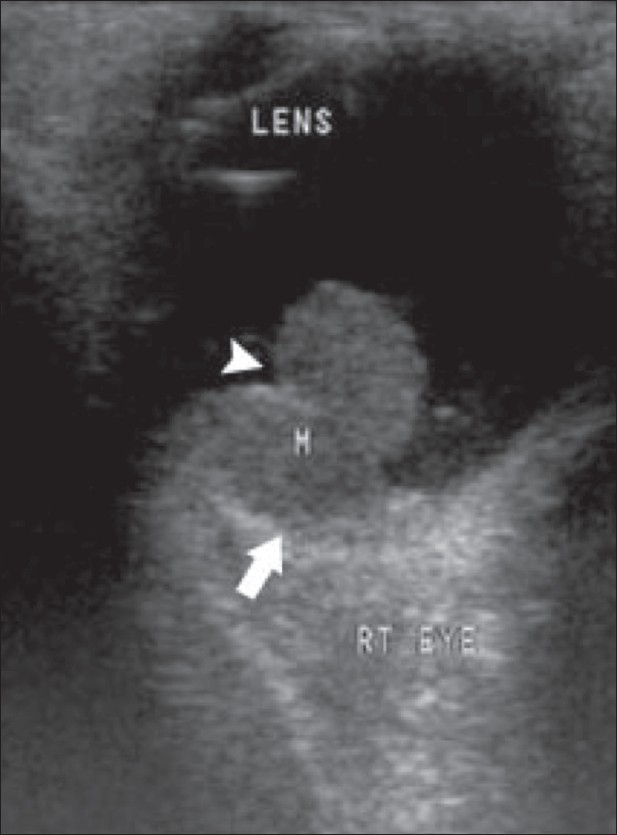
Choroidal melanoma. The same case as in Figure 11 shows a tumor, with choroidal excavation (arrow). The tumor shows a bilobed or cottage-loaf appearance, which is caused by waisting (arrowhead) as it breaks through Bruch's membrane

**Figure 13:**
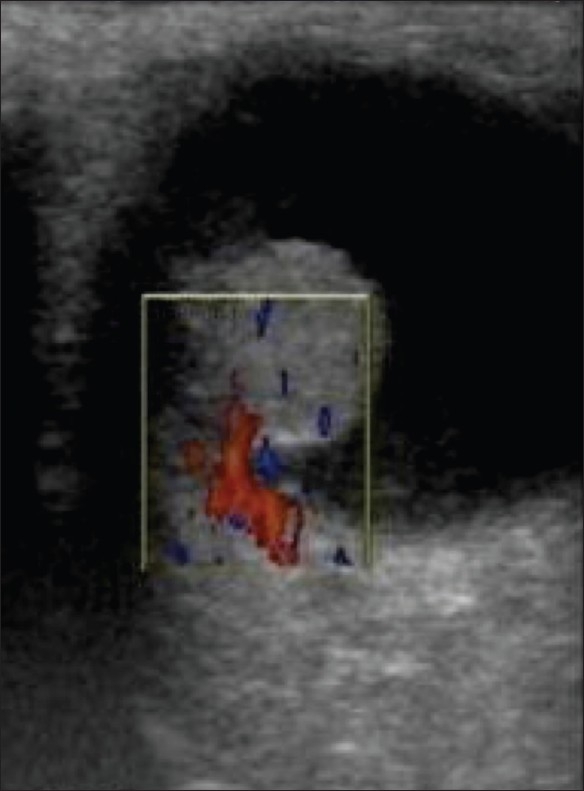
Choroidal melanoma. Color Doppler reveals vascularity in a malignant choroidal melanoma

Choroidal melanoma, the commonest primary intraocular tumor in adults, arises from the choroid and ciliary body. Most of these lesions arise posterior to the equator of the eyeball. On B-scan, it is seen as a lenticular-shaped mass arising from the choroid. USG is used to assess scleral erosions and extraocular extension into orbital fat.[1–3,6] Some tumors have a collar-button or mushroom shape. Blood flow within the tumor is seen on colour Doppler as pulsating channels or lakes of colour. Choroidal melanoma may be associated with retinal detachment. The tumor has a bilobed or ‘cottage-loaf’ appearance, which is caused by waisting as it breaks through Bruch's membrane. The tumor usually demonstrates choroidal excavation. Colour Doppler reveals the vascularity of the lesion[1–6] [Figure 11–13].

### Retinoblastoma

**Figure 14:**
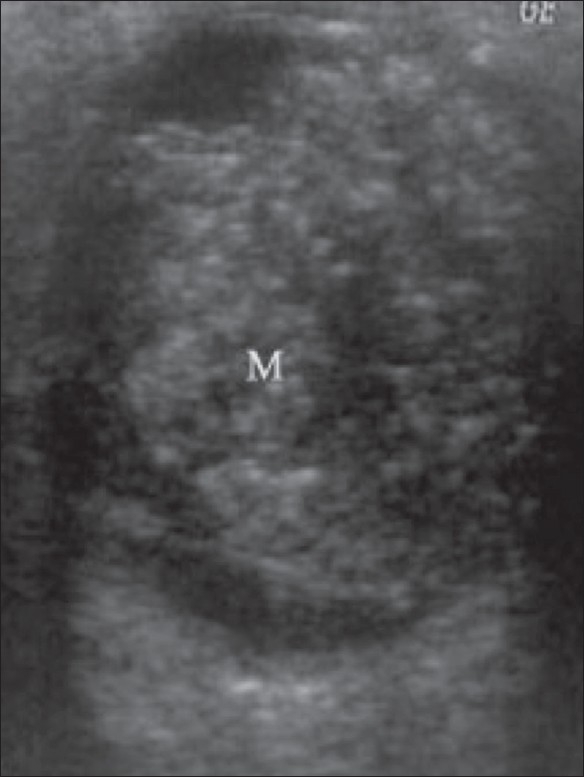
Retinoblastoma. This is the case of a child with leukocoria. B-scan reveals a hyperechoic tumor (M) extensively filling the posterior segment. The calcium deposits, seen as highly reflective foci, are pathognomonic. The tumor outline is irregular

**Figure 15 (A,B):**
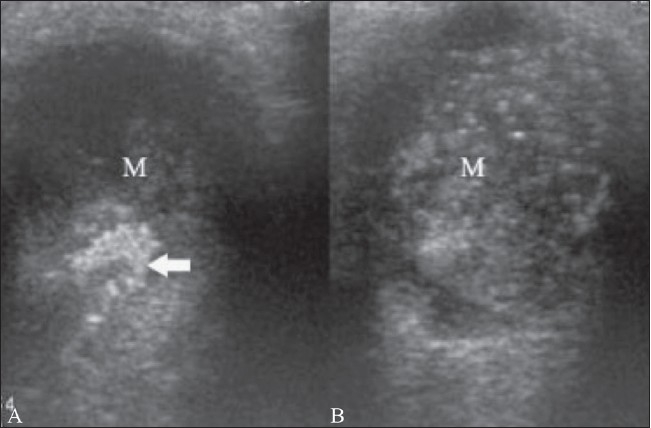
Retinoblastoma. This patient has bilateral retinoblastoma. On B-scan, the tumor (M) in the left eye (A) shows an echogenic focus (arrow), suggestive of calcification. A similar mass (M) is seen in the right eye (B)

Retinoblastoma is the commonest primary intraocular tumor of childhood. It arises from the embryonic retinal epithelium of the primary optic vesicle. It is usually unilateral but may be bilateral in one-third of cases. It presents in childhood as leukocoria.[1–5,7,8] The tumor projects from the retina into the vitreous chamber. Some tumors produce subretinal lesions and cause retinal detachment. Calcium deposits are commonly seen within the tumor. The calcium deposits, which are seen as highly reflective foci, are pathognomonic of the condition.[1,2] The tumor outline is irregular. B-scan may help in the detection of optic nerve invasion resulting from extraocular spread of the tumor[1–5,7] [Figures 14 and 15].

### Phthisis bulbi

**Figure 16 (A,B):**
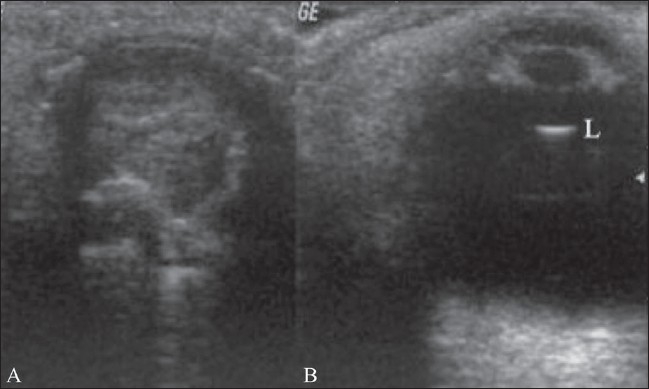
Phthisis. The left eyeball is clinically blind. The B-scan of the left eye (A) shows a shrunken globe with extensive calcification and loss of the normal shape. There was a history of trauma. The normal right eyeball (B) is shown for comparison

Phthisis bulbi is an end-stage condition following ocular trauma and hemorrhage. The eye is blind, small, and nonfunctioning, with extensive calcification.[Figure 16] There is loss of the normal ocular shape.[1,4]

### Ocular trauma

**Figure 17:**
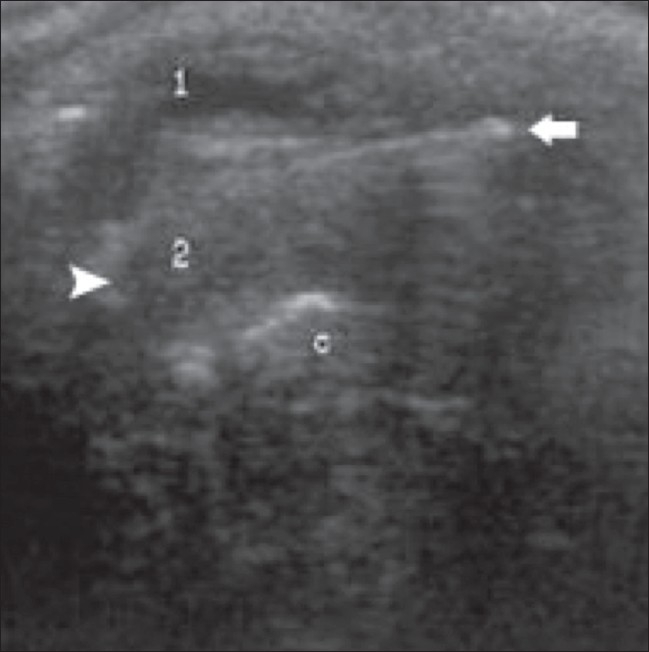
Penetrating injury. USG reveals vitreous hemorrhage, rupture of the globe and shrinkage of the globe due to loss of vitreous as the result of a penetrating injury. A foreign body (arrow) is seen crossing the eyeball (arrowhead). 1 - anterior segment, 2 - posterior segment, 3 - calcification

Ocular trauma either due to blunt injury or penetrating injury can result in vitreous hemorrhage. There may be rupture and shrinkage of globe due to loss of vitreous as a result of a penetrating injury. There is distortion of normal ocular shape and intravitreal hemorrhage develops with or without concomitant posterior vitreous detachment.[1–3] The foreign body that caused the injury may be seen within the eyeball, for example a metallic object in a missile injury or blasts. Sometimes penetrating foreign bodies like a wooden object or a metallic rod may be seen crossing the eyeball. Metallic foreign bodies show a posterior reverberation artifact[1–3] [Figure 17].

### Vitreous detachment

**Figure 18 (A,B):**
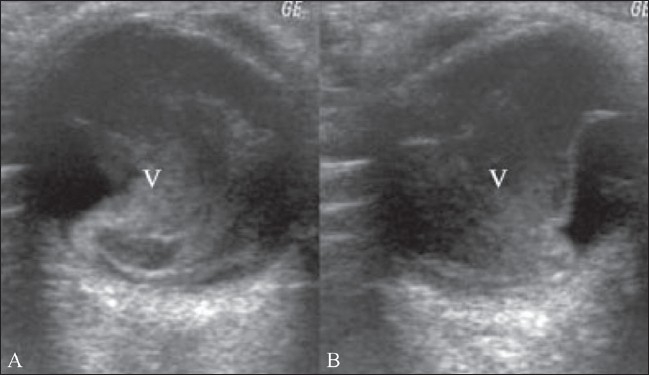
Vitreous detachment. B-scan shows a reduction in the volume of the vitreous gel (V). On movement, the vitreous shows increased mobility with a mirror image configuration, when the eye is deviated to one side (A) and then to the other (B)

Vitreous detachment is seen in elderly individuals. It results from gel liquefaction and collection of fluid in the subvitreal space, which in turn, results in vitreous detachment. It is seen frequently in cataractous eyes on B-scan. B-scan shows reduced volume of vitreous gel. USG also shows marked mobility and elasticity of the detached vitreous, with a mirror image configuration when the eye is deviated to one side and then to the other[1–4] [Figure 18].

### Vitreous hemorrhage

**Figure 19:**
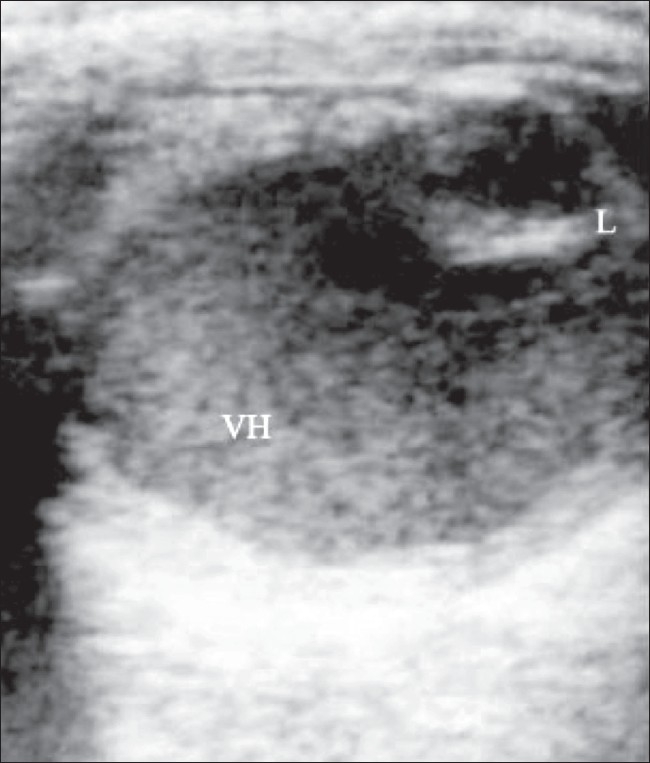
Vitreous hemorrhage. B-scan of the right eye reveals widespread low-intensity echoes in the vitreous chamber, which show marked after-movement on dynamic scanning. The normal lens (L) is seen anteriorly

Vitreous hemorrhage can result from tearing due to vitreoretinal traction, diabetic retinopathy, vasculitis, subarachnoid hemorrhage, and blunt trauma to the eye. The presence of blood cells in the vitreous gives rise to low-intensity echoes. Later, the hemorrhage may organize and develop fibrinous membranes. Figure 19 shows a B-scan of the right eye which reveals widespread low-intensity echoes in the vitreous chamber, with marked after-movement on dynamic scanning.[1–4]

## Conclusion

1. B-scan is a reliable, safe, cheap, and rapid investigation.
2. The 7.5-MHz probe provides excellent quality real-time imaging.
3. B-scan helps in evaluating the posterior segment in the presence of opaque ocular media.[1,2]
4. B-scan is useful for preoperative planning.[3]
5. B-scan is the preferred screening modality in extraocular lesions.[1,2]
6. It is a feasible option in rural centers.
